# Mechanistic Investigations of the Mitochondrial Complex I Inhibitor Rotenone in the Context of Pharmacological and Safety Evaluation

**DOI:** 10.1038/srep45465

**Published:** 2017-04-04

**Authors:** Sabrina Heinz, Alexius Freyberger, Bettina Lawrenz, Ludwig Schladt, Gabriele Schmuck, Heidrun Ellinger-Ziegelbauer

**Affiliations:** 1 Bayer AG, Drug Discovery, Pharmaceuticals, Wuppertal, Germany

## Abstract

Inhibitors of the mitochondrial respiratory chain complex I are suggested to exert anti-tumor activity on those tumors relying on oxidative metabolism and are therefore of interest to oncology research. Nevertheless, the safety profile of these inhibitors should be thoroughly assessed. Rotenone, a proven complex I inhibitor, has shown anti-carcinogenic activity in several studies. In this context rotenone was used in this study as a tool compound with the aim to identify suitable biomarker candidates and provide enhanced mechanistic insights into the molecular and cellular effects of complex I inhibitors. Rats were treated with 400 ppm rotenone daily for 1, 3 or 14 consecutive days followed by necropsy. Classical clinical endpoints, including hematology, clinical chemistry and histopathology with supporting investigations (FACS-analysis, enzymatic activity assays) were examined as well as gene expression analysis. Through these investigations, we identified liver, bone marrow and bone as target organs amongst approx. 40 organs evaluated at least histopathologically. Our results suggest blood analysis, bone marrow parameters, assessment of lactate in serum and glycogen in liver, and especially gene expression analysis in liver as useful parameters for an experimental model to help to characterize the profile of complex I inhibitors with respect to a tolerable risk-benefit balance.

Rotenone is a lipophilic, naturally occurring compound, mainly derived from the roots and stems of *Lonchocarpus* and *Derris* species. It had been widely used as pesticide and piscicide, however was then withdrawn from the market in many countries^1^ due to its toxicity. Rotenone acts as a strong inhibitor of complex I of the mitochondrial respiratory chain (MRC). The mechanism of action (MOA) comprises inhibition of electron transfer from the iron-sulfur centers in complex I to ubiquinone, leading to a blockade of oxidative phosphorylation with limited synthesis of ATP^2^. Furthermore, incomplete electron transfer to oxygen could lead to the formation of reactive oxygen species (ROS). This rotenone-induced ROS production, with an assumed damage of mitochondria components, including mitochondrial DNA, can eventually lead to apoptosis^3^,^4^. In addition to the effects on the MRC, several studies have demonstrated that rotenone inhibits microtubule assembly independently of a specific energy-requiring step through tubulin binding, resulting in mitotic arrest and inhibition of cell proliferation^5^,^6^,^7^. Due to these MOAs and the high lipophilicity, enabling rotenone to easily cross biological membranes including the blood–brain barrier^8^, rotenone became interesting for Parkinson’s disease (PD) research. Defective mitochondrial function, especially decreased complex I activity and increased oxidative stress, has been demonstrated in a subset of patients with PD^9^,^10^. Due to their important role in neuronal polarity, axonal transport and synaptic plasticity, microtubule dysfunction may also play a role in PD progression^11^,^12^. Moreover, rotenone exposure has been shown to correlate with the occurrence of several PD-like symptoms in humans^13^. Chronic administration of rotenone has caused selective degeneration of nigral dopaminergic neurons with histopathological hallmarks of PD and PD-like locomotor symptoms in animal models^14^. Therefore, rotenone-based PD models have been developed for investigating behavioral syndromes and molecular mechanisms as well as screening novel anti-parkinsonian drugs and diagnostic methods^15^. In addition to rotenone´s neurotoxic effect, it has been suggested that rotenone has anti-carcinogenic activity. Rotenone has been known to induce apoptosis and inhibit cell proliferation of various human cancer cell lines^16^,^17^,^18^,^19^,^20^, to inhibit spontaneously and chemically induced liver tumors in mice^21^,^22^,^23^, and chemically induced colon tumors^24^ and tongue tumors in rats^25^. Despite these results suggesting a possible anti-tumorigenic activity of rotenone, its neurotoxic effects may exclude its use as an anti-carcinogenic compound. However, other complex I inhibitors such as metformin, an antidiabetic drug, have also been suggested to exert anti-tumor activity on those tumors relying on oxidative metabolism^26^. Accordingly, complex I inhibitors are of interest in oncology research. In this context we performed a systemic study in rats with rotenone as a tool compound measuring classical clinical endpoints, including hematology, clinical chemistry and histopathology, with additional supporting investigations, like FACS-analysis and enzymatic activity assays. Several of these parameters have been measured before in rotenone studies by others. We still included these to obtain quantitative results in our setting for direct comparison with those measured for the first time in our study. The focus was mainly on organs and tissues with high proliferative activity, e.g. the hematopoietic system, and with high metabolic activity, e.g. the liver. Furthermore we performed gene expression analysis to examine additional pathways and functions affected by rotenone at the molecular level. In addition to the liver, the heart and brain stem were chosen for gene expression profiling due to their high energy demand and the known neurotoxic effect of rotenone. By using this experimental design we intended to identify MOA-based biomarkers and provide enhanced mechanistic insights into the action of complex I inhibitors to improve the assessment of compounds in drug development.

## Results

### General toxicological findings

The treatment of male rats with 400 ppm rotenone through the diet (resulting in a daily intake of 52.5 mg/kg body weight) led to a reduced body weight compared to the control group. After a decrease of around 10% within the first 2 days of treatment, body weight remained constant over the 2 week study. This resulted in a 33% lower weight at terminal necropsy (14d) compared to the weight increasing control group. Correspondingly, absolute organ weights of rotenone exposed animals were significantly reduced compared to control animals, which was especially the case for liver and kidneys. For a complete overview of body and organ weights see Supplementary Fig. S1 and Table S1. Histopathological investigations indicated no changes in kidneys, heart and brain, amongst many other organs evaluated. However, liver, hematopoietic tissue and bone were identified as primary target organs by histopathological and other supporting investigations like gene expression profiling, hematology and FACS-analysis. Detailed findings are described below.

### Complex I inhibition

**Figure 1:**
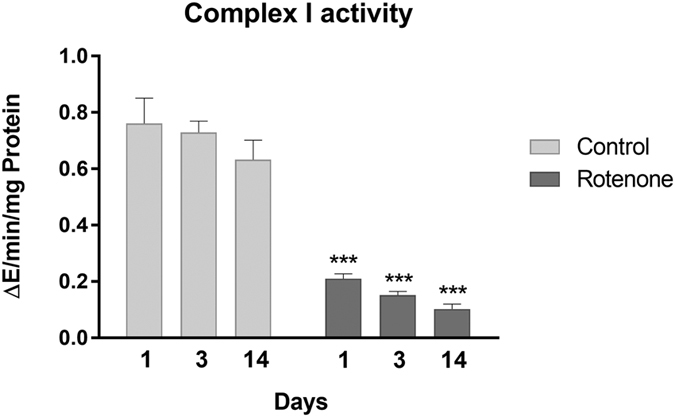
Complex I inhibition. Complex I activity in isolated mitochondria from the livers of control and rotenone treated animals (400 ppm) is shown as mean with SD (n = 5) after the three treatment durations. Statistical significance (Two-Way ANOVA with Sidak multiple comparison test) is indicated by ***P < 0.001 compared to time-matched control groups.

To determine the potency of complex I inhibition by rotenone after the different treatment durations, complex I activity was measured enzymatically in isolated liver mitochondria. Rotenone exposure induced a strong and significant decrease in complex I activity (Fig. 1), which was around 70–80% at every time point compared to the time matched control group (see Supplementary Fig. S2), indicating that the inhibition by rotenone was maintained even after isolation of the mitochondria.

### Changes in liver after rotenone treatment

**Figure 2:**
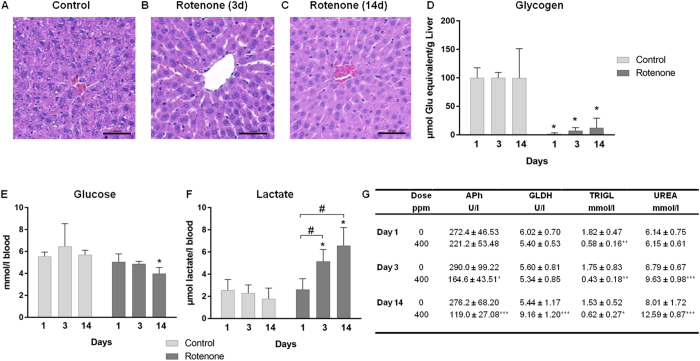
Changes in the liver after rotenone treatment. (**A**–**C**) Histopathological changes in the liver of rats treated with 400 ppm rotenone for 3 (**B**) and 14 days (**C**) compared to control (**A**). Shown are representative hematoxylin and eosin stained liver sections with a scale bar of 50 μm. (**D**) The enzymatically determined decrease of glycogen content in livers is shown as mean with SD (n = 5) at the three different time-points. (**E**) Decreased blood glucose, (**F**) time dependent increased blood lactate concentrations and (**G**) other significant blood parameters (alkaline phosphatase (APh), glutamate dehydrogenase (GLDH), triglyceride (TRIGL), urea (UREA)) are also presented as mean with SD (n = 5) at the three different time-points. Statistical analysis was performed with Two-Way ANOVA with Sidak multiple comparison test. Statistical significance is indicated by *P < 0.05, **P < 0.01, and ***P < 0.001 compared with time-matched control groups or by ^#^P < 0.05 for the factor “time” of one treatment group.

Histopathological findings in the liver (summarized in Table 1) revealed a distinct loss of glycogen in hepatocytes and condensation of the cytoplasm, most noticeably after 1 and 3 days of rotenone treatment (Fig. 2B). In addition, decreased granulation of the rough endoplasmic reticulum was observed, accompanied by substantial alterations in cytoplasmic morphology. These changes were also found in the 14-day treatment group. The histopathologically observed glycogen loss was consistent with an enzymatically determined significant decrease of the glycogen content in the livers of rotenone treated relative to control animals at every time point (Fig. 2D). In addition, triglycerides (TRIGL) were significantly decreased on all measurement days (Fig. 2G), and blood glucose was decreased after two weeks (Fig. 2E), whereas blood lactate concentration was significantly increased in a time-dependent manner (Fig. 2F). Overall, this suggests a hypocaloric status induced by rotenone exposure. Together, the liver-associated alterations summarized above, and increased urea and glutamate dehydrogenase (GLDH) and decreased alkaline phosphatase (APh) levels (Fig. 2G), indicate that rotenone predominantly affects metabolic processes in the liver. For a complete overview of clinical chemistry parameters see Supplementary Table S2.

### Effects of rotenone on hematopoietic tissue

**Figure 3:**
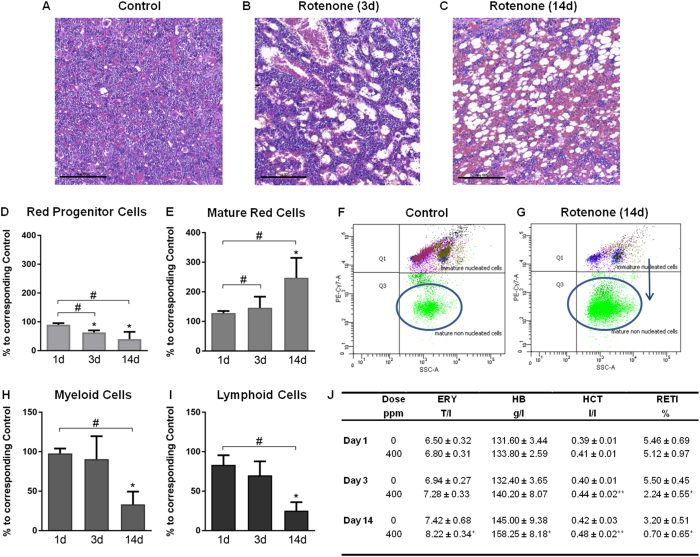
Rotenone effects on hematopoietic tissue. (**A**–**C**) Histopathological changes in the bone marrow of rats treated with 400 ppm rotenone for 3 (**B**) and 14 days (**C**) compared to control (**A**), indicating decreased bone marrow cellularity and an increase in adipocytes after 3 days of treatment (**B**), with increased severity after 14 days (**C**). Shown are representative hematoxylin and eosin stained bone marrow sections with a scale bar of 200 μm. (**D**–**G**) FACS results of femoral bone marrow, including (**D**) red progenitor cells, (**E**) mature red blood cells, (**H**) myeloid cells and (**I**) lymphoid cells are shown as percent change (mean with SD, n = 5) at the three different time-points relative to the corresponding time-matched control. (**F**,**G**) Representative FACS plots of immature nucleated cells and mature non nucleated cells (blue circle) of a control animal (**F**) and a rotenone treated animal after 14 days (**G**). The blue arrow indicates a shift towards more mature erythroid forms. (**J**) Significantly affected blood cell parameters are presented as mean with SD (n = 5) at the three different time-points. Statistical analysis was performed with Two-Way ANOVA with Sidak multiple comparison test.

Histopathological investigations of the bone marrow in the femur and sternum showed subtle dilation and hyperemia of blood sinuses merely after 1 day of treatment. Bone marrow adipocytes started to increase after 3 days of dosing, indicating bone marrow depletion (Fig. 3B), which further increased by two weeks leading to a spongy atrophy of the marrow (Table 1 and Fig. 3C). One primary target of rotenone seemed to be erythropoiesis. In the spleen, extramedullary erythropoiesis was reduced in all animals after 3 days of treatment (Table 1). After 14 days, it was observed in all treated animals again with moderate severity, but also in three of five control animals with minimal severity. The latter may be related to the age of the animals used here, since extramedullary hematopoiesis declines with increasing age in rats^27^. FACS-analysis of femoral bone marrow indicated a significant increase in the erythroid cell lineage with a shift towards more mature forms over time (Fig. 3D–G). In addition, red progenitor cells, myeloid and lymphoid cells were significantly diminished notably after 14 days of rotenone treatment, confirming general bone marrow depletion (Fig. 3H+I).

The effects of rotenone on hematopoietic cells overall were investigated through the examination of red and white blood parameters and thrombocytes in blood. Significant increases in erythrocyte count, hemoglobin concentration and hematocrit were observed after 14 days in animals treated with rotenone compared to the control group (Fig. 3J). Moreover the number of reticulocytes was significantly decreased after 3 days in rotenone treated rats (Fig. 3H). These findings confirm the histopathological and FACS observations, revealing an effect of rotenone on erythropoiesis. For a complete overview of hematology parameters see Supplementary Table S3.

### Effects of rotenone on bone

**Figure 4:**
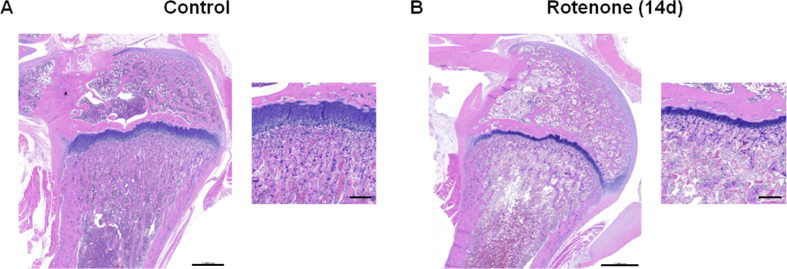
The effect of rotenone on bone. (**A**+**B**) Histopathological changes in the bone of rats administered with 400 ppm rotenone for 14 days (**B**) compared to control (**A**). Shown are representative hematoxylin and eosin stained bone sections of the femur with a higher magnification of the growth plates (scale bar of 1000 μm and 200 μm, respectively).

In the femur and tibia, minor changes in the growth plate were already encountered after 3 days of rotenone treatment. Dilation of subchondral blood sinuses, a slightly reduced height of the physis and reduction of spongiosa formation in the subchondral plate indicated the beginning of growth arrest and atrophy of the growth plate. After 14 days of treatment, a distinct suppression of the proliferative and hypertrophic zone of the cartilage was visible leading to a thin growth plate, cessation of primary enchondral ossification in the subchondral plate and a decrease in subsequent secondary ossification. No regressive changes were noted, and the growth zones rather resembled those in older rats, but with less trabecular stability in the subchondral plate (Fig. 4B). These histopathological findings are summarized in Table 1.

### Gene expression analysis

**Figure 5:**
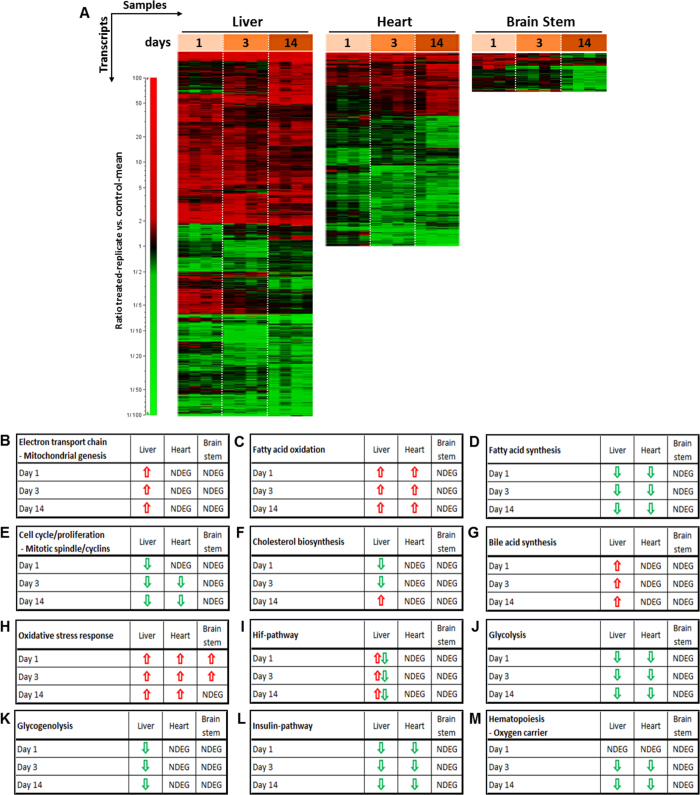
Gene expression analysis. (**A**) Heatmaps represent expression profiles of transcripts significantly affected by rotenone treatment in the liver, heart (left ventricle apex) and brain stem over time. Each column represents a dataset from one organ sample (n = 4), and each row represents a transcript. Changes in gene expression are demonstrated by the color bar to the left of the diagram. Red represents increased and green represents decreased expression levels, indicated as ratios relative to the mean of the time-matched control group. (**B**–**M**) Tables show different affected pathways. Arrows represent the direction of deregulation of genes associated with the respective pathway. NDEG (not found as deregulated by our selected cutoffs) indicates that genes belonging to this pathway were not significantly deregulated in the specific organ and time point indicated.

Gene Expression Analysis was performed in several organs to examine pathways and functions affected by rotenone at the molecular level. The liver and heart were chosen due to the high metabolic activity and the high energy demand, respectively. For the brain, brainstem was selected as subregion, since it has been shown that Lewy bodies, the hallmark lesions of degenerating neurons in the brains of patients with Parkinson’s disease (PD)^28^, first appear in the olfactory bulb, medulla oblongata and pontine tegmentum, the letter two belonging to the brain stem^29^. Accordingly, in our short term study we expected gene expression changes rather in the brain stem as compared to other brain regions. After the analysis of the whole transcriptome, the strongest rotenone-induced effects on gene expression were observed in liver (1444 deregulated genes) compared to heart (650 deregulated genes) and brain stem (52 deregulated genes) (Fig. 5A). This finding supports the results reported above, proposing the liver as a primary target organ of rotenone. Only a small number of genes were deregulated in the brain stem after rotenone treatment compared to the liver and heart (Fig. 5A). For interpretation the deregulated genes were assigned to different biochemical categories and subcategories in the context of the main biological functions (a complete overview of genes assigned to such categories is given in Supplementary Table S4). The major functions (Fig. 5B–M) represented by the deregulated genes indicate, in line with the pharmacological action, increased expression levels of genes associated with mitochondrial genesis and the mitochondrial electron transport chain. Particularly genes encoding mitochondrial complex I subunits were up-regulated primarily in the liver (Fig. 5B). An increased expression of genes associated with fatty acid oxidation (Fig. 5C) and decreased expression of genes involved in fatty acid synthesis (Fig. 5D) and cell cycle/proliferation (Fig. 5E) were observed in liver and heart. Concerning the latter, genes encoding mitotic spindle components were down-regulated. Genes encoding cholesterol biosynthesis enzymes were initially down-regulated after 1 and 3 days of rotenone treatment, but were subsequently up-regulated after 14 days (Fig. 5F). However, several genes associated with bile acid synthesis were up-regulated throughout in the liver tissue (Fig. 5G). Genes known to be induced in response to oxidative stress were up-regulated in the liver, heart and brain stem (Fig. 5H). Interestingly, increased mRNA levels of the gene encoding *Sat2 (spermidine/spermine N1-acetyltransferase family member 2*), involved in degradation of the hypoxia-inducible factor 1-alpha (Hif1α) were observed in the liver at all time points. However, Hif1 target genes and *Hyou1 (Hypoxia up-regulated 1*), usually induced by hypoxia, were down-regulated (Fig. 5I). A rather unexpected down-regulation of genes encoding glycolysis enzymes in the liver and heart (Fig. 5J) and genes encoding glycogenolysis enzymes and regulators in liver were detected (Fig. 5K). In addition, the expression level of the gene encoding *GAA* (Lysosomal alpha-glucosidase), which is essential for breakdown of glycogen to glucose in lysosomes, was increased in the liver. Furthermore, genes belonging to the insulin pathway were down-regulated in the liver and heart (Fig. 5L). Interestingly genes associated with hematopoiesis and the oxygen carrier hemoglobin were down-regulated after 3 and 14 days of treatment in the liver and heart (Fig. 5M). Another interesting finding was the up-regulation of *Igfbp2 (insulin-like growth factor binding protein 2*) mRNA after all durations of treatment and the down-regulation of *Igf1 (insulin-like growth factor 1*) mRNA after 3 and 14 days. These two genes play a role in bone formation. Further implications of the gene deregulations described above will be discussed below.

## Discussion

Rotenone acts as a strong inhibitor of the mitochondrial complex I. The resulting incomplete electron transfer within the MRC leads to ATP depletion and in turn promotes the formation of ROS and thereby induces oxidative stress and apoptosis in cells^2^,^3^. Moreover, rotenone can inhibit microtubule assembly through binding to tubulin leading to the inhibition of cell proliferation^7^.

In oncology drug research mitochondrial metabolism has recently evolved as a target for cancer therapy, especially for tumors relying on oxidative metabolism^26^, with complex I suggested as one possible site of action. Since rotenone has also shown anti-carcinogenic activity in several studies^21^,^22^,^23^,^24^, the aim of the present study was to identify biomarker candidates and provide enhanced mechanistic insights into the molecular and cellular effects of complex I inhibitors after *in vivo* treatment, using rotenone as a tool compound. Therefore rats were exposed to 0 or 400 ppm rotenone through their diet up to 14 days and various parameters including gene expression profiling were examined. Three target organs, liver, bone marrow and bone, were identified through these investigations.

### Rotenone triggers a hypocaloric status

Complex I inhibition by rotenone was confirmed in the liver through measurement of complex I activity in isolated mitochondria after 1, 3 and 14 days of treatment. Gene expression analysis in liver revealed increased expression of mRNAs encoding proteins associated with mitochondrial genesis and the mitochondrial electron transport chain, especially of those encoding complex I subunits, after rotenone treatment. This could be a feedback reaction to complex I inhibition by rotenone, likely leading to reduced mitochondrial energy supply. It is proposed that this reduced supply was partially responsible for the weight loss within the first 2 days and the subsequent constant but not increasing body weight development observed during the 14 day treatment period.

Additionally, in the liver, heart and brain stem a number of genes involved in oxidative stress responses were up-regulated. This was potentially caused by an increase in the formation of ROS upon incomplete electron transfer due to the inhibition of complex I activity by rotenone, as previously described^4^. In parallel, increased expression of the gene encoding *Sat2 (spermidine/spermine N1-acetyltransferase family member 2*), which plays a role in the degradation of hypoxia-inducible factor 1-alpha (Hif1α), was observed in the liver of rotenone treated rats. In addition, expression of Hif1 target genes e.g. *Lox (lysyl oxidase*) and *Loxl2 (lysyl oxidase-like 2*) and the gene *Hyou1 (Hypoxia up-regulated 1*), usually induced by hypoxia, was decreased. Hif1 is a heterodimeric transcription factor consisting of two subunits Hif1-α and Hif1-β and regulates cellular responses to low oxygen. Under normoxic conditions, Hif1-α is hydroxylated by oxygen-dependent prolyl hydroxylases (PHD) leading to its degradation via ubiquitination. Under hypoxic conditions, PHDs are inactive, thus Hif1-α becomes stabilized, dimerizes with Hif1-β and can induce transcription of its target genes^30^,^31^,^32^,^33^. Although activation of Hif1 in the context of hypoxia is suggested to be induced by mitochondrial ROS formation^34^,^35^, complex I inhibitors, including rotenone, have been known to inhibit the Hif1-pathway even under hypoxic conditions^26^,^36^,^37^. This might be explained by reduced O_2_ consumption in mitochondria with an increase in cytosolic oxygen levels due to complex I inhibition and subsequent reduced electron transport chain activity. This could result in greater availability of oxygen for PHDs, in turn leading to Hif-1α degradation.

This apparent reduced energy supply may also be responsible for other findings in liver. Histopathological investigations and direct glycogen measurements indicated early loss of liver glycogen within the first 3 days. Rotenone-treatment induced liver glycogen depletion was observed previously by others^38^. From these observations one may expect increased glycogenolysis leading to the release of glucose into the bloodstream, allowing energy generation through the glycolysis pathway. However, gene expression analysis showed mostly decreased expression of genes encoding proteins participating in glycogenolysis. This could potentially be explained by a negative feedback reaction resulting from the earlier increased degradation of glycogen. An exception to the mostly decreased expression of genes encoding glycogenolysis-associated proteins was an increased hepatic expression of the gene for *GAA* (Lysosomal alpha-glucosidase). *GAA* is essential for the breakdown of glycogen to glucose in lysosomes, suggesting all available resources of glycogen were utilized. Lactate, which is a component in the glycogen-glucose metabolic network, was increased in the blood after rotenone administration. Gene expression deregulation in this context indicated decreased expression of genes coding for proteins involved in the insulin pathway and for glycolytic enzymes in the liver and heart. Downregulation of glycolysis may be explained by feedback regulation to high blood lactate levels to avoid severe lactic acidosis. Blood glucose levels were only slightly reduced after two weeks of treatment presumably due to a tight regulation by the rat to maintain stable blood glucose levels. Further, the rat is able to avoid hypoglycemia through the modulation of metabolic pathways that generate glucose from other resources including the breakdown of glycogen, proteins, and lipids. This suggests the metabolic regulation network is supported by results from other blood parameters including increased urea and decreased triglyceride levels potentially resulting from increased protein and lipid/fatty acid degradation, respectively. The latter is supported by the increased expression of genes encoding fatty acid oxidation enzymes in the liver and heart. The decreased expression levels of genes coding for proteins involved in cell proliferation and fatty acid synthesis at all time points in liver and heart, may potentially represent a response to reduced mitochondrial energy supply overall, to avoid not acutely required energy consuming processes. This includes e.g. the mRNA for *Srebf1 (Sterol regulatory element binding transcription factor 1*), which is the major regulator of fatty acid enzyme gene transcription.

With respect to cholesterol biosynthesis, the expression of genes encoding the corresponding enzymes were decreased after 1 and 3 days, but then increased after 14 days of rotenone treatment. However, genes encoding bile acid synthesis enzymes were increased at all time points analyzed. This may be explained by stimulated increased synthesis of bile acids required as emulsifiers for dietary lipids in the intestine, leading to decreased cholesterol levels, in turn activating cholesterol synthesis at the later time point. Supporting evidence for this mRNA levels encoding *Srebf2 (Sterol regulatory element binding transcription factor 2*), the major regulator of cholesterol synthesis gene transcription, were increased at the 14 day time point. In addition, increased mRNA levels were also seen for the gene encoding the key enzyme of cholesterol biosynthesis *Hmgcr (3-hydroxy-3-methylglutaryl-CoA reductase*), and others of this biosynthetic pathway.

### Rotenone causes bone marrow atrophy and influences erythropoiesis

Depletion of the bone marrow as observed by histopathological analysis of the femur and sternum, started already after the first day of treatment, leading to marked atrophy after 2 weeks. Furthermore, FACS investigations showed a decrease of red progenitor cells, myeloid and lymphoid cells especially after 14 days of treatment, confirming general bone marrow depletion. Bone marrow atrophy was also observed after 13 weeks of oral rotenone administration of 300 ppm up to 1200 ppm through the diet in the NTP technical report (1988)^39^.

Further, rotenone treatment had distinct effects on erythropoiesis, leading to decreased extramedullary erythropoiesis in the spleen, as detected histopathologically. FACS-analysis indicated increased numbers of erythroid cells in femoral bone marrow with a shift towards more mature forms over time. This shift to more mature erythroid stages in the bone marrow was paralleled in peripheral blood by an increase in red blood parameters (Ery, Hb, Hct) at the later time point, and a decrease of the number of reticulocytes, i.e. early immature erythroid cells, already on day 3 after rotenone treatment, in line with and as an indicator for the developing bone marrow depletion. In parallel to reduced numbers of reticulocytes, gene expression analysis showed decreased expression of genes encoding isoforms of the oxygen carrier hemoglobin in the heart and liver tissue. Further, decreased expression of genes involved in hematopoiesis mainly in the heart after 3 and 14 days, was observed. This likely reflects rotenone-induced processes in blood circulating through these organs.

Together, these results suggest a significantly impaired generation of hematopoietic cells. In general, hematopoietic tissue has a strong energy demand due to its high proliferative activity. Therefore mitochondrial complex I inhibition by rotenone leading to decreased energy supply could be a likely reason for bone marrow depletion. The additional inhibitory effect of rotenone on microtubule assembly^5^,^6^,^7^ may also be involved in some of the observed effects, leading to mitotic arrest and inhibition of cell proliferation of these rapidly dividing cells. Previously, diminished pyrimidine synthesis was discussed as a potential reason for ineffective hemopoiesis after rotenone treatment^40^ with the resulting depletion of nucleotides leading to reduced cell division and proliferation. In this context we observed increased mRNA levels of the gene encoding uridine phosphorylase 2 (*Upp2*) in the liver, which is involved in the salvation pathway of pyrimidine bases for nucleotide synthesis. Although not analyzed by gene expression, such genes may also be deregulated in hematopoietic tissue and may contribute to ineffective hemopoiesis. Furthermore, the assumed inhibition of the Hif1-pathway by rotenone, as described above, could be involved in bone marrow atrophy, as Hif1 is required for maintenance of the hematopoietic stem cell pool. Thus, deletion of Hif-1α promotes the differentiation and exhaustion of hematopoietic stem cells^41^.

The acute transient increase in erythrocytes may furthermore be explained as a feedback reaction to eryptosis. This particular form of apoptosis seen only with erythrocytes can be induced by oxidative stress and energy depletion^42^, both known effects of rotenone. In addition, treatment of erythrocytes with rotenone *in vitro* leads to eryptosis^43^. However, no increase in bilirubin, a breakdown product of eryptosis, was observed in the blood in the present study. Thus, the significance of eryptosis for our observations remains unclear. Although this potential mechanism has been insufficiently characterized and does not fit completely with all observations made in this study, it can be summarized, that after the onset of treatment, an acute mobilization of red blood cells occurs, followed by the development of severe bone marrow depletion. These events can be explained by both known MOAs of rotenone, i.e. inhibition of complex I activity and of microtubule assembly.

### Rotenone treatment leads to bone growth suppression

In the femur and tibia, suppression of bone growth could be observed starting after 3 days of dosing and increasingly over the 2-week study period. The growth arrest became evident in the proliferation and hypertrophic zones of the epiphyseal growth plate. This could be caused by the observed bone marrow atrophy due to the fact that osteoblasts and osteoclasts, both required for bone remodeling, are differentiated from bone marrow cells. Osteoblasts, which are essential for bone reassembly, develop from bone marrow mesenchymal stem cells^44^,^45^. Additionally, osteoclasts, crucial for bone disassembly, are differentiated from macrophage precursors in the hematopoietic lineage in response to the cytokine Receptor Activator of NF-κB Ligand (RANKL)^46^. In this context, direct inhibition of osteoclast differentiation through down-regulation of RANKL is described for rotenone *in vitro*^47^.

Another potential reason for the rotenone-induced bone growth suppression in our study could be an induction of *Igfbp2 (insulin-like growth factor binding protein 2*), which was detectable in our gene expression data at all three time points, with highest levels after 14 days of treatment., Moreover, *Igf1 (insulin-like growth factor 1*)-mRNA was down-regulated after 3 and 14 days. It has been shown, that overexpression of *Igfbp2* reduces bone mass in growth hormone transgenic mice^48^. In addition, Jehle *et al*.^49^ observed a positive correlation between high serum levels of IGFBP-2 and bone loss in women and men. Furthermore, Amin and colleagues (2004)^50^ reported that serum IGFBP-2 levels were inversely associated with bone mass in humans. Conversely, proliferation and differentiation of growth plate osteoblasts and chondrocytes could be stimulated by IGF-I^51^,^52^,^53^. Hence, a reduced bone density could be shown in IGF-I-knockout-mice^54^. Moreover, it has been considered that IGFBP-2 is a negative regulator for IGF-I-induced bone formation^55^. Thus, our observations, i.e. induction of *Igfbp2* mRNA and reduction of *Igf1* mRNA, suggest involvement of these gene deregulations in bone growth suppression. Other than this, the growth arrest in the proliferation zone in our study could also simply be explained with an overall growth reduction as a response to reduced energy storage, e.g. fat and glycogen.

The fibroblast growth factor 21 (FGF21)-pathway may represent a further mechanisms for negative regulation of bone mass, as it has previously been involved in the inhibition of osteoblastogenesis and enhancement of marrow adipogenesis^56^. *FGF21* expression is induced in the liver through PPARα activation^57^,^58^ and in white adipose tissue through PPARγ activation^59^,^60^,^61^. Further, Kim *et al*.^62^ demonstrated the ability of rotenone to induce *FGF21 in vitro* due to the stress response associated with ROS production^62^. However, our gene expression analysis in the liver, although exhibiting up-regulation of *PPARα* mRNA after one day of rotenone treatment, did not reveal increased expression of *FGF21* mRNA. The idea that *PPARy* mediated *FGF21* expression is induced by rotenone within adipose tissue can be neither assumed nor ruled out and would warrant further investigation.

### Administration of rotenone through the diet did not lead to obvious neurotoxicity

The high lipophilicity of rotenone enables it to cross all biological membranes easily, including the blood-brain barrier^63^. Nevertheless, histopathological investigations of the whole brain, which was sampled and processed according the STP Position Paper by Bolon *et al*.^64^ to identify the different brain regions, including substatia nigra^64^, could not detect rotenone-induced neurotoxicity in our study, as previously described for other rotenone studies. Further, gene expression analysis in brain stem identified a small number of genes with increased expression levels. A subset of these genes are known to be involved in an oxidative stress response which may be related to neurotoxicity. This rather weak effect in the brain in our study may be explained by the administration method used. Rotenone has limited bioavailability^65^. It is only incompletely absorbed in the stomach and intestine and is efficiently metabolized in the liver. Thus, high brain concentrations can only be achieved by parenteral administrations and presumably not by the oral route. The absence of extensive neurotoxicity after oral exposure is also consistent with the fact, that rats in the chronic two years study from Marking (1988)^66^ did not develop any behavioral disorders or neuropathological effects at doses 30 times greater than in the rotenone-based PD model with a systemic infusion performed by Betarbet and colleagues (2000)^14^. In addition, no adverse effects in the brain were reported in the NTP technical report (1988)^39^ after 13 weeks of oral rotenone administration up to 1200 ppm and after two years up to 75 ppm in male F344/N rats. Therefore the administration route reported for rotenone-based PD models is mainly parenteral^15^, whereas administrations via the oral route as used in our study usually do not induce clear neurotoxic effects.

### General considerations of toxicological compared to potential pharmacologic effects in cancer

In two-year rat NTP studies with up to 75 ppm rotenone survival was not affected, yet 14 day studies with higher doses indicated dose-dependent reduced body weight gain (50–600ppm) and body weight loss starting at 1200 ppm in 14-days studies. Our 14-day rat study with 400ppm suggested metabolic effects which may be related to positive effects in certain cancers (e.g. inhibition of fatty acid synthesis and cell cycle/proliferation), yet were accompanied by toxicological phenotypes including bone marrow atrophy and the overall hypocaloric status of the rats. However, although concentration ranges up to 75 ppm may be tolerated for a life time of the rat, it is unclear whether these doses are sufficient to induce anti-cancer responses. This is supported by observations of usually steep dose-response-curves of mitochondrial electron transport chain inhibitors^67^ (and unpublished observations), suggesting that lowering the dose to some extent may already reduce toxicity, but in parallel also decrease potential anti-cancer activity.

## Conclusions

In summary, the treatment of rats with rotenone administered through the diet induced an overall hypocaloric status, which was quite obvious in the liver, and characterized by e.g. glycogen depletion. Although this finding in general was already reported for rotenone in previous studies, our gene expression analysis, especially in the liver, adds new mechanistic details for the characterization of this status. In addition, our investigations provide new insights into the effect of rotenone on hematopoietic tissue and bone. Moreover, the lack of neurotoxicity despite the ability of rotenone to easily pass the blood-brain barrier indicates that the hematopoietic tissue and the bone are more sensitive to rotenone than the brain, in case of oral applications. In case complex I inhibitors were administered by routes allowing efficient brain exposure, the brain may be a preferential target, and absence of changes in the biomarkers suggested here would not exclude more sensitive effects in the brain. In these cases potential toxicity to dopaminergic neurons would need to be investigated in more details as described for Parkinson’s disease models^68^,^69^. Although previously reported investigations suggest possible anti-carcinogenic effects of rotenone, the toxic effects described by others and in this study, including bone marrow depletion and bone atrophy, exclude its use as a safe anti-cancer compound. However, other complex I inhibitors such as metformin, a drug developed originally as an antidiabetic, have been suggested to exert anti-tumor activity on tumors relying on oxidative metabolism^26^. The safety profile of such compounds must still be rigorously assessed. Our experimental model with multiple time points, including bone marrow parameters, blood analysis, glycogen assessment in liver, lactate assessment in serum, and in particular gene expression analysis in liver, in comparison to rotenone and potentially other reference compounds, should help to characterize this profile.

## Methods

### Animal studies

All animal experiments were performed according to the German guidelines for care and use of laboratory animals and were approved by the State Agency for Nature, Environment and Consumer Protection North Rhine-Westphalia in Germany (LANUV). Male RccHan Wistar rats aged 7–8 weeks were randomly assigned to the vehicle or treatment group (n = 5). All animals were housed under controlled standard conditions (12 h light and 12 h dark cycle, at 22 ± 2 °C) and received food and water *ad libitum*. 0 or 400 ppm rotenone (Sigma Aldrich, Steinheim, Germany) was administered through the diet for 1, 3 or 14 consecutive days, followed by necropsy. The diet was prepared by mixing 400 ppm of pure rotenone powder into the chow (V1534–0 ssniff R/M-H) using a mixing granulator (Loedige, Paderborn, Germany). Based on the food intake and body weight data, the rotenone intake was calculated as 52.5 mg/kg body weight per day (Pristima^®^). The individual animal body weights were determined daily and the food consumption was measured weekly. Clinical examinations of all animals were performed once and inspections on mortality and morbidity twice daily. On the day of necropsy, animals were sacrificed by exsanguination under isoflurane anesthesia. Blood was collected for clinical chemistry and hematology and several organs were removed, weighed, aliquoted, fixed in formalin or flash frozen in liquid nitrogen and stored at −80 °C, described further below.

### Preparation of mitochondria

Mitochondria were isolated as described by Fernández-Vizarra and colleagues (2006)^70^ with slight modifications. Frozen liver (500 mg) was disrupted in homogenization medium (0.075 M sucrose; 0.225 M mannitol; 1 mM EGTA; 0.01% BSA; adjusted to pH 7.4) using a loosely-fitting potter homogenizer (Potter S, B. Braun Biotech International) at 1500 rpm with 20 strokes up and down. The homogenate was centrifuged at 1000 × g for 10 min at 4 °C. The supernatant was transferred into a fresh tube and centrifuged at 12000 × g for 6 min at 4 °C. The resulting pellet was washed in homogenization medium and re-centrifuged at 12000 × g for 6 min at 4 °C. This pellet containing the mitochondrial fraction was solubilized in MAITE buffer (25 mM sucrose; 75 mM sorbitol; 100 mM KCl; 0.05 mM EDTA; 5 mM MgCl_2_; 10 mM Tris-HCl; 10 mM H_3_PO_4_; adjusted to pH 7.4) and centrifuged once more at 12000 × g for 6 min at 4 °C. The final pellet was resuspended in 500 ml MAITE buffer. All steps above were performed on ice. Mitochondrial protein concentration was measured using the Pierce™ BCA Protein Assay Kit (Thermo Scientific, Rockford, USA) with bovine serum albumin as standard. The mitochondria were subsequently snap frozen in liquid nitrogen and stored at −80 °C until further use. All chemicals were purchased from Sigma Aldrich, Steinheim, Germany.

### Measurement of complex I activity

Complex I (NADH ubiquinone reductase) activity was measured according to Birch-Machin and colleagues (1989)^71^ by recording NADH oxidation by complex I using the coenzyme Q10 analogue decylubiquinone (dUb) as an electron acceptor. 300 μg of mitochondrial protein was mixed with 320 μl reaction-solution (20 mM KH_2_PO_4_ (pH 7.2), 5 mM MgCl_2_, 0.65 mg/ml KCN, 25 mg/ml defatted BSA, 0.5 mg/ml antimycin), 4.19 mg/ml dUb and either DMSO or 2.5 mM rotenone in a final volume of 950 μl. After incubation for 2 min the reaction was started by addition of 1.92 mg/ml NADH. Oxidation of NADH was determined by following the decrease in absorbance of NADH at 340 nm for 2 min with a spectrophotometer. Complex I activity was calculated using the difference between the measured rates in the absence (DMSO only) vs. presence of rotenone. All chemicals were purchased from Sigma Aldrich, Steinheim, Germany.

### Histopathological analysis

On the day of necropsy several organs and tissues (including liver, heart, kidneys, brain, spleen, femur and sternum with bone marrow) were fixed in 10% buffered formalin, embedded in paraffin (Paraplast^®^, Carl Roth GmbH & Co. Kg, Karlsruhe, Germany), sectioned (approximately 3 μm) and stained with hematoxylin and eosin (HE). The brain was sampled and processed according the STP Position Paper by Bolon *et al*.^64^ and the different brain regions were identified by using the brain atlas of Paxinos and Watson (1997)^72^. All sections were examined by light microscopy and histopathological findings were graded by a semi-quantitative severity scoring system (grade 1 - minimal; grade 2 - slight; grade 3 - moderate; grade 4 - marked; grade 5 - severe). Histopathological findings were entered online into the PathData^®^ software version 6.2c2 (Pathology Data Systems, Inc., Mt. Arlington, NJ, USA).

### FACS-analysis

Flow cytometric analysis (FACS) of rat bone marrow was performed as described by Saad *et al*.^73^ with slight modifications. Briefly, during necropsy the left femur was removed and trimmed, distal epiphyses were cut off, bone marrow tissue was flushed out with HBSS 1x +Ca/Mg (Hank´s Balanced Salt Solution; Thermo Fisher Scientific, Waltham, USA), suspended and filtered. The adjusted bone marrow cell suspension (1 × 10^6^ cells/tube) was washed with ice-cold phosphate buffered saline (PBS, Alfa Aesar, Ward Hill, USA) containing 0.5% bovine serum albumin (BSA, Sigma Aldrich, Steinheim, Germany)(PBS/BSA) and centrifuged at 1200 rpm for 10 min at 4 °C. Cells were first incubated for 20 min in the dark at 4 °C with 5 μl of fluorescein isothiocyanate (FITC)-conjugated mouse anti-rat CD45 (leucocyte common antigen) and 10 μl of phycoerythrin (PE)-conjugated mouse anti-rat CD71 (transferrin receptor antigen) monoclonal antibodies (Becton Dickinson, Franklin Lakes, USA) and subsequently washed with ice-cold PBS/BSA and centrifuged at 1200 rpm for 5 min at 4 °C. The resulting cell pellet was resuspended in 400 μl ice-cold PBS/BSA. 20 μl of LDS-751 staining solution (a cell-permeate nucleic acid stain; Life Technologies, Carlsbad, USA) was added and tubes were incubated for 30 min in the dark at 4 °C. Cells were subsequently washed with ice-cold PBS/BSA, centrifuged at 1200 rpm for 5 min at 4 °C and resuspended in 400 μl ice-cold PBS/BSA. FACS-analysis was performed with a FACSCanto II flow cytometer and FACSDiva Software (Becton Dickinson, Franklin Lakes, USA) with data collection from 10000 cells. The following bone marrow cell populations were determined: lymphoid, myeloid and nucleated erythroid cells. In addition to the workflow by Saad *et al*.^73^ another parameter was added to discriminate between reticulocytes (CD71-positive cells) and mature erythroid cells (CD71-negative cells).

### Glycogen determination in liver

Frozen liver (approximately 1 g) was used for enzymatic determination of the glycogen content using the amyloglucosidase-method described by Keppler and Decker (1974)^74^.

### Hematology and clinical chemistry analysis

On the day of necropsy blood samples were taken from the Vena jugularis of non-anesthetized animals for glucose and lactate determination. Blood samples for determination of all other parameters were collected during necropsy from the retro-bulbar venous plexus.

Determination of all major hematological parameters was conducted using the Hematology System ADVIA 2120i (Siemens Healthcare Diagnostics GmbH, Eschborn, Germany).

Serum clinical chemistry parameters including alanine aminotransferase (ALAT), aspartate aminotransferase (ASAT), alkaline phosphatase (APh), gamma-glutamyltransferase (gamma-GT), lactate dehydrogenase (LDH), creatine kinase NAC (CK), cholesterol (CHOL), triglycerides (TRIGL), creatinine (CREA), urea, total bilirubin (Bili-t), protein, albumin and glucose from deproteinized whole blood were measured with the ADVIA 2400 Analyzer (Siemens Healthcare Diagnostics GmbH, Eschborn, Germany). Serum glutamate dehydrogenase (GLDH) and lactate from deproteinized whole blood were measured with the Cobas c501 Analyzer (Roche Diagnostics GmbH, Heiligenhaus, Germany).

### RNA extraction

Total RNA was isolated from liver (70 mg) using the RNeasy Mini Kit; from heart (left ventricle apex, 30 mg), using the RNeasy Fibrous Tissue Mini Kit and from brain stem (70 mg) using the RNeasy Lipid Tissue Mini Kit, all according to the manufacturer’s instructions (Qiagen, Hilden, Germany). RNA quality was determined using the Bioanalyzer^®^ 2100 and RNA 6000 Nano Kits (Agilent, SantaClara, CA) and quantity was assessed using a Nanodrop^®^ 1000 Spectrophotometer.

### Gene expression profiling

Starting with 300 ng high quality total RNA from the liver, heart and brain stem of 4 replicate animals per treatment group, biotin labelled cDNA fragments for hybridization on the GeneChip^®^ Rat Transcriptome Array (RTA) 1.0. (Affymetrix; Santa Clara, USA) were prepared according to the manufacturer’s instructions. Using the Affymetrix GeneChip Scanner 3000, fluorescent images of the GeneChips were generated. The resulting CEL files were pre-processed by applying the Guanine Cytosine Count Normalization (GCCN) correction and the Signal Space Transformation (SST) algorithm developed by Affymetrix. Subsequently the Robust Multi-array Average (RMA) condensing method was used to obtain a single expression value for each transcript. The RTA 1.0 array covers 194,000 transcripts. Significantly deregulated genes were identified with Genedata^®^ Analyst by using N-Way-ANOVA based on the factors treatment (liver: P < 0.000001; heart: P < 0.00001; brain stem: P < 0.001), time (liver: P < 0.0001; heart: P < 0.0001; brain stem: P < 0.001) and treatment + time (liver: P < 0.0001; heart: P < 0.001; brain stem: P < 0.001), and with a Kruskal-Wallis-test (P < 0.05 for all organs) between treated and control groups combined with a 1.8-fold deregulation cut-off.

The significance levels for N-Way-ANOVA were adjusted differently between organs to identify the most dominant changes in gene expression yet to obtain a reasonable number of genes for a detailed analysis. Ingenuity Pathway Analysis (IPA; Qiagen; Hilden, Germany) was used for further investigations.

### Statistics

Statistical calculations for data shown in bar diagrams and tables were performed using Graphpad Prism (Vers.6). Statistical significance for the factors time and treatment were determined using a Two-Way ANOVA with the Sidak multiple comparison test. For each time point the data are represented as mean + SD from five replicate animals per treatment group (control; rotenone). Statistics for microarray analysis were performed as described in the ‘*Gene expression profiling’* section.

## Additional Information

**How to cite this article:** Heinz, S. *et al*. Mechanistic Investigations of the Mitochondrial Complex I Inhibitor Rotenone in the Context of Pharmacological and Safety Evaluation. *Sci. Rep.* **7**, 45465; doi: 10.1038/srep45465 (2017).

**Publisher's note:** Springer Nature remains neutral with regard to jurisdictional claims in published maps and institutional affiliations.

## Supplementary Material

Supplementary Dataset 1

Supplementary Dataset 2

## Figures and Tables

**Table 1:** Major histopathological findings.

Organ: finding	Treatment duration	Score	Control	Rotenone
Liver: cytoplasmic change/atrophy	1 day	incidence	0	5
		severity	—	3.2
	3 days	incidence	0	5
		severity	—	2.8
	2 weeks	incidence	0	5
		severity	—	3.6
Bone marrow femur: hyperemia/sinus dilation	1 day	incidence	0	2
		severity	—	0.8
	3 days	incidence	0	5
		severity	—	2.0
	2 weeks	incidence	0	5
		severity	—	3.2
Bone marrow femur: depletion/increased adipocytes	1 day	incidence	0	1
		severity	—	0.4
	3 days	incidence	0	2
		severity	—	0.6
	2 weeks	incidence	0	5
		severity	—	3.4
Bone marrow femur: atrophy	1 day	incidence	0	0
		severity	—	—
	3 days	incidence	0	0
		severity	—	—
	2 weeks	incidence	0	5
		severity	—	3.0
Spleen: reduced extramedullary hemopoiesis (erythropoiesis)	1 day	incidence	0	0
		severity	—	—
	3 days	incidence	0	5
		severity	—	3.0
	2 weeks	incidence	3	5
		severity	1.2	3.0
Bone, femur: atrophy/arrest growth plate	1 day	incidence	0	0
		severity	—	—
	3 days	incidence	0	4
		severity	—	1.6
	2 weeks	incidence	0	5
		severity	—	3.0

Incidence (number of animals; n = 5 per treatment group) of rotenone-caused histopathological changes in rats, after the three different treatment periods. The “severity” score indicates the mean severity from grading 1–5, representing minimal-slight-moderate-marked-massive, respectively.
