# Supplementary material for: Mechanistic Investigations of the Mitochondrial Complex I Inhibitor Rotenone in the Context of Pharmacological and Safety Evaluation
Source: Sci Rep. 2017 Apr 4;7:45465. doi: 10.1038/srep45465 (PMC5379642; doi:10.1038/srep45465)
Supplement: Supplementary Dataset 1 [file srep45465-s1.doc]

SUPPLEMENTARY MATERIAL

Mechanistic Investigations of the Mitochondrial Complex I Inhibitor Rotenone in the Context of Pharmacological and Safety Evaluation

Sabrina Heinz, Alexius Freyberger, Bettina Lawrenz, Ludwig Schladt, Gabriele Schmuck and Heidrun Ellinger-Ziegelbauer


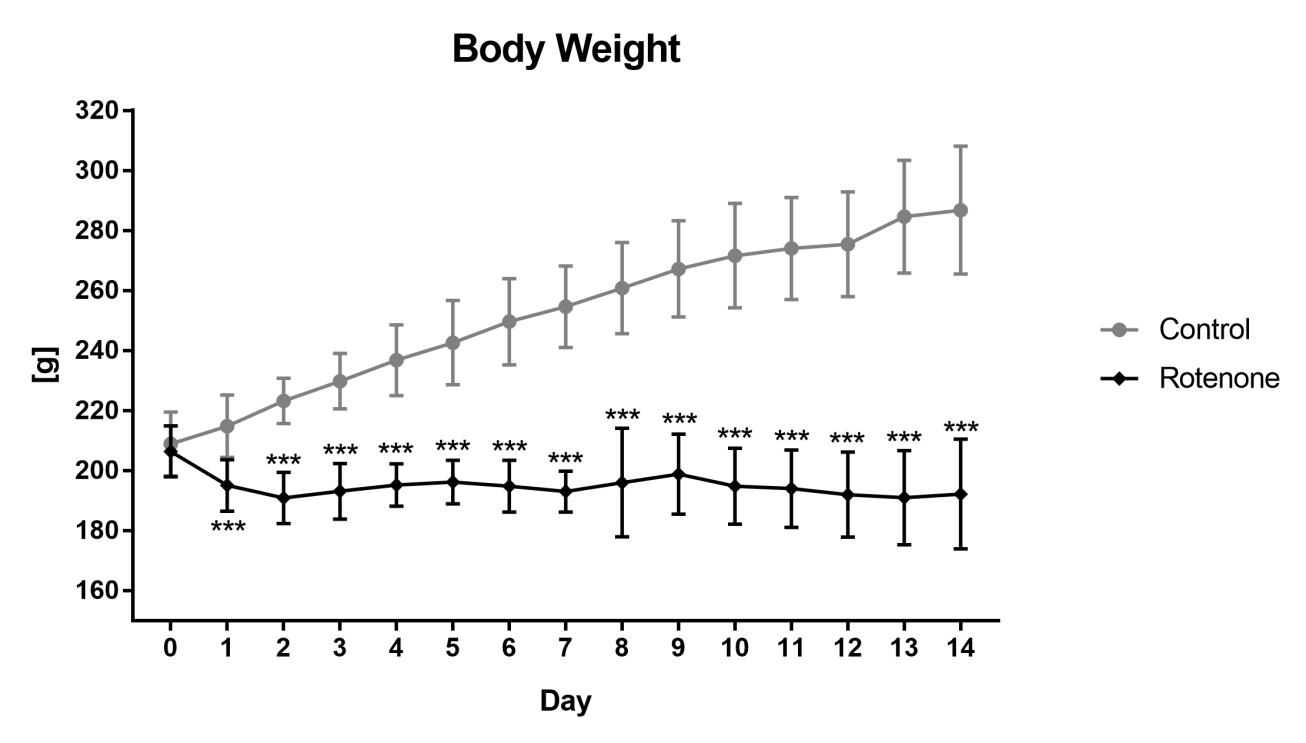


Fig. S1: Body weight after rotenone treatment. Development of body weights of control and rotenone treated animals (400 ppm) is shown as mean with SD (day 0 and 1: n=15; day 2 and 3: n=10; day 4-14: n=5). Statistical significance (Two-Way ANOVA with Sidak multiple comparison test) is indicated by ***P<.001 compared to time-matched control groups.


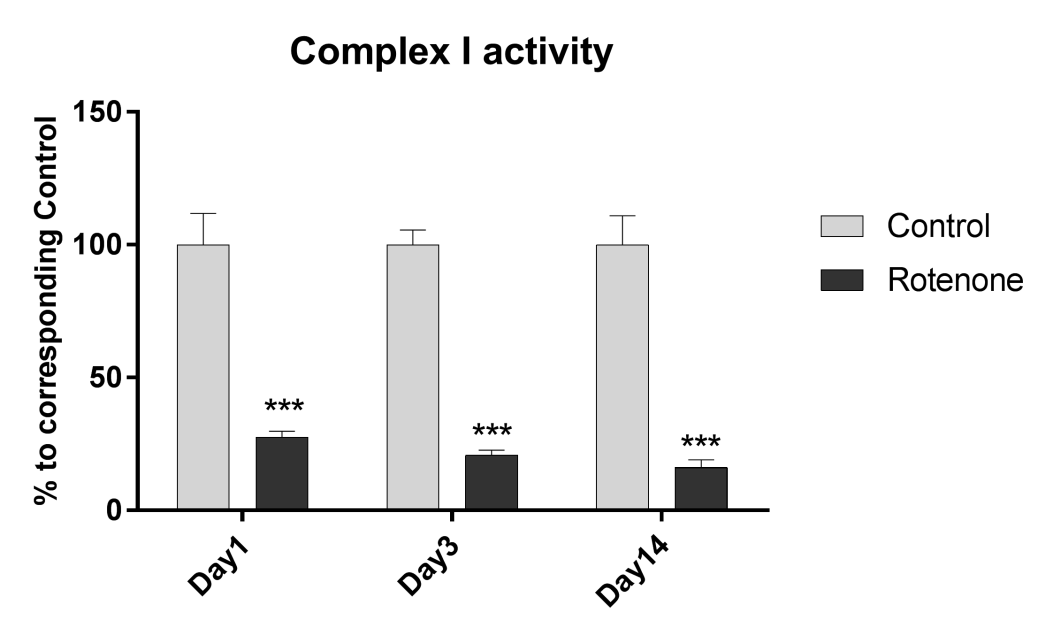


Fig. S2: Complex I inhibition. Complex I activity in isolated mitochondria from livers of control and rotenone treated animals (400 ppm) is shown in % to the corresponding control group with SD (n=5) after the three treatment durations. Statistical significance (Two-Way ANOVA with Sidak multiple comparison test) is indicated by ***P<.001 compared to time-matched control groups.

Table S1: Absolute and relative organ weights. Mean values with SD (n=5) of absolute and relative organ weights of control and rotenone treated animals after the three treatment durations. Body weight changes in treated animals are shown in % compared to time-matched control groups. Statistical significance (Two-Way ANOVA with Sidak multiple comparison test) is indicated by ***P<.001 compared to time-matched control groups.

Table S2: Overview of clinical chemistry parameters (Alanine Aminotransferase (ALAT); Aspartate Aminotransferase (ASAT); Alkaline Phosphatase (APh); Glutamate Dehydrogenase (GLDH); gamma-Glutamyl Transferase (gamma-GT); Lactate Dehydrogenase (LDH); Creatine Kinase (CK); Glucose; Cholesterol (CHOL); Triglycerides (TRIGL); Creatinine (CREA); Urea; Bilirubin (Bili-t); Lactate; Protein; Albumin). Mean values with SD (n=5) of clinical chemistry parameters of control and rotenone treated animals after the three treatment durations. Statistical significance (Two-Way ANOVA with Sidak multiple comparison test) is indicated by **P<.01 and ***P<.001 compared to time-matched control groups.

Table S2 continued

Table S3: Overview of hematology parameters (Erythrocytes (ERY); Hemoglobin (HB); Hematocrit (HCT); Mean Corpuscular Hemoglobin (MCH); Mean Corpuscular Hemoglobin Concentration (MCHC); Mean Cell Volume (MCV); Reticulocytes (RETI); Thrombocytes (THRO); Leucocytes (LEUCO); Lymphocytes (LYM); Neutrophils (NEUTRO); Basophils; Eosinophils (EOS); Monocytes (MONO); Atypical). Mean values with SD (n=5) of hematology parameters of control and rotenone treated animals after the three treatment durations. Statistical significance (Two-Way ANOVA with Sidak multiple comparison test) is indicated by *P<.05 and **P<.01 compared to time-matched control groups.

Table S3 continued
